# Enhancing PQQ production in *Acinetobacter calcoaceticus* through uniform design and support vector regression

**DOI:** 10.3389/fmicb.2025.1556322

**Published:** 2025-08-07

**Authors:** Yu-han Li, Su-hang Yao, Yan Zhou, Xiu-lan He, Zhe-ming Yuan, Qiu-long Hu, Cheng-wen Shen, Xin Li, Yuan Chen

**Affiliations:** ^1^National Research Center of Engineering and Technology for Utilization of Botanical Functional Ingredients, Hunan Agricultural University, Changsha, China; ^2^Hunan Province Microbiology Research Institute, Changsha, China; ^3^Central South University Graduate School Long Ping Branch, Changsha, China; ^4^Yuelushan Laboratory of Hunan Province, Changsha, China; ^5^Hunan Engineering & Technology Research Centre for Agricultural Big Data Analysis & Decision-Making, Hunan Agricultural University, Changsha, China; ^6^Hunan Engineering Research Center for Endophytic Microbial Resources Mining and Utilization, Changsha, China

**Keywords:** *Acinetobacter calcoaceticus*, PQQ, uniform design, support vector regression, formulation optimization

## Abstract

A novel machine learning-assisted approach for formula optimization, termed UD-SVR, is introduced by combining uniform design with support vector regression. This method was employed to optimize both the formulation and fermentation conditions for pyrroloquinoline quinone (PQQ) production by *Acinetobacter calcoaceticus*. In just two rounds of 66 experimental treatments, UD-SVR effectively optimized a formulation involving eight factors at the shake-out level scale, enhancing PQQ production from 43.65 mg/L to 73.40 mg/L—an impressive 68.15% increase. Notably, the optimized formulation is also cost-effective, featuring minimized consumption of pivotal elements like carbon and nitrogen sources. The machine learning-supported UD-SVR method presents an inclusive resolution for optimizing experimental designs and analyses in multi-factor, multi-level formulations, characterized by robust guidance, lucid interpretability, and heightened efficiency in optimization.

## 1 Introduction

Pyrroloquinoline quinone (PQQ), stands as a pivotal cofactor among the oxidoreductases residing on bacterial cell membranes (Duine and Frank, 1980). Beyond its involvement in enzymatic catalysis during redox reactions, PQQ assumes a critical role in electron transfer mechanisms (Song et al., 2023), showcasing robust antioxidant characteristics (Gao et al., 2004; Willner et al., 2007). Its profound impacts extend to promoting metabolic activities, growth, development, and fostering resilience (Yao et al., 2024; Rucker et al., 2021). It represents a novel biocomposite material within the industrial domain, offering unparalleled safeguarding for biomolecules (Liang et al., 2015).

Presently, the primary approaches for PQQ production encompass chemical synthesis and microbial fermentation (Choi et al., 2008). Despite the higher production achieved through chemical synthesis, this method is encumbered by intricate synthesis steps, elevated production costs (Paz et al., 1996; Qin et al., 2022), difficulties in managing by-products, low product recovery rates, and environmental pollution concerns. In contrast, microbial fermentation stands out for its straightforward process, gentle reaction conditions, cost-effectiveness, and enhanced productivity (Ke et al., 2021), establishing itself as the predominant method for industrial-scale PQQ production in contemporary times (Ren et al., 2023). The current forefront of PQQ production via microbial fermentation revolves around the selection of superior high-production strains, as well as the optimization of culture media compositions and fermentation condition (Liu et al., 2020; Luna and Boiardi, 2008). For instance, utilized artificial neural networks and response surface methodology to fine-tune the culture media components for the *Methylobacillus* sp. Zju323 strain. Their investigation pinpointed cobalt chloride hexahydrate, magnesium sulfate heptahydrate, and p-Aminobenzoic acid as three pivotal factors significantly impacting PQQ production, resulting in a notable 35% increase in PQQ production post-optimization. Furthermore, Zhang et al. (2022) employed a hybrid approach combining atmospheric pressure room temperature plasma mutagenesis with high-throughput screening, successfully identifying a mutant strain exhibiting heightened PQQ production, enhancing PQQ production from 31.4 mg/L to 48.4 mg/L.

*Acinetobacter calcoaceticus* (*A. calcoaceticus*) is a Gram-negative bacterium in the family *Moraxellaceae* of the class *Gammaproteobacteria*. It is strictly aerobic, grows at temperatures ranging from 20°C to 35°C, and is widely distributed in soil, water, wastewater, and food. It also exhibits notable halotolerance and plant growth–promoting properties (Oteino et al., 2015). Our laboratory previously isolated an *A. calcoaceticus* strain capable of fermentative PQQ production. Notably, its PQQ biosynthetic pathway requires only four genes, compared with six in *Klebsiella pneumoniae* and seven in *Methylobacterium extorquens* AM1, representing a streamlined system for cofactor synthesis (Schwarzenbacher et al., 2004; Puehringer et al., 2008). Moreover, *A. calcoaceticus* exhibits broad substrate specificity and high product purity, utilizing carbon sources such as ethanol, methanol, glycerol and polyols. These characteristics underscore the organism’s strong potential for fermentative PQQ production. However, under standard media compositions and fermentation conditions, PQQ production reached only 43.65 mg/L. Optimization of fermentation parameters is therefore essential to enhance yield; yet traditional one-factor-at-a-time experiments are time-consuming, costly and fail to capture interactive effects, and although orthogonal and uniform designs address some interactions, their low resolution limits efficacy (Duine and Frank, 1980; Yuan et al., 2007). To overcome these constraints, machine-learning–assisted methods have been increasingly applied to fermentation optimization (Sharma and Mishra, 2023; Xu et al., 2025). Support vector machines (SVM) (Che et al., 2017), in particular, excel with small datasets, prompting us to develop a uniform design–support vector regression (UD-SVR) strategy for medium and process parameter optimization. This innovative technique, rooted in machine learning principles, optimized eight key fermentation parameters encompassing carbon and nitrogen levels, fermentation temperature, pH, among others, tailored specifically for *A. calcoaceticus*. Following two iterative cycles comprising 66 optimization trials, the PQQ fermentation production surged to 73.40 mg/L, showcasing a substantial 68.15% enhancement over the initial production levels.

## 2 Materials and methods

### 2.1 Strain and medium

The PQQ-producing *A. calcoaceticus CDWB36, i*solated and characterized by our laboratory, is securely preserved in a − 80°C freezer, encapsulating its biological essence for further scientific exploration.

Liquid LB medium: peptone 10.0 g/L, yeast powder 5.0 g/L, sodium chloride 10.0 g/L, pH 7.0 ± 0.2. Solid LB medium: liquid LB medium supplemented with 15.0 g/L agar. Initial fermentation medium: yeast powder 10.0 g/L, anhydrous ammonium sulfate 2.0 g/L, L-glutamic acid 1.0 g/L, L-tyrosine 1.0 g/L, Na_2_HPO_4_ 2.0 g/L, KH_2_PO_4_ 1.4 g/L, MgSO_4_·7H_2_O 1.0 g/L, calcium chloride 0.4 g/L, trace elements solution 0.4 g/L, pH adjusted to 7.0 ± 0.2, inoculum volume 1%, temperature 28°C. Trace elements solution: FeSO_4_·7H_2_O 80.0 mg/L, ZnSO_4_·7H_2_O 22.5 mg/L, NaCl 15.0 mg/L, KI 0.3 mg/L, H_3_BO_3_ 3.0 mg/L, CuSO_4_ 5.0 mg/L.

### 2.2 Bacterial culture and fermentation

The strain was introduced into LB medium and cultured at 28°C for 24 h. A small portion was subsequently transferred into 150 mL Erlenmeyer flasks containing 50 mL of LB liquid fermentation medium for continued activation. After24 hours of shaking at 180 rpm and 28°C, the fermentation medium was inoculated with 1% of the activated culture and incubate at 180 rpm and 28°C for 7 days.

### 2.3 Measurement method for PQQ production

Using spectral analysis (Letokhov, 1975), we detected various concentrations (1, 2, 5, 10, 20, 25, 50, 100 mg/L) of PQQ standard solutions. To eliminate interference, the absorbance value of the blank fermentation medium was subtracted during sample testing. A standard curve was established with PQQ concentration on the *x*-axis and OD_330_ on the *y*-axis (Supplementary Figure S1).

For cultures subjected to different fermentation conditions, each treatment was sampled three times, with each replicate measured in duplicate. Subsequently, 1 mL of fermentation liquid was centrifuged at 4°C and 12,000 rpm for 2 min. The supernatant was then collected, and the absorbance at 330 nm (OD_330_) was measured using a Hitachi UV3000 spectrophotometer. The PQQ concentrations were calculated by averaging the OD_330_ values from three replicates, based on the standard curve.

### 2.4 Optimization process of UD-SVR

In the optimization process, we used the hybrid-level module of DPS software to generate a uniform design for the experimental designs. The experiments were then conducted, and the PQQ productions under various treatments were measured. Next, nonlinear feature screening with an SVR model was conducted to identify the key factors influencing PQQ production. Based on the retained factors, an SVR prediction model was trained to forecast PQQ production across all possible treatment combinations (Xiao et al., 2021). Frequency-based statistical optimization was subsequently applied to the predicted values to determine the optimal level for each factor. Depending on the outcome, either a new round of uniform design was initiated or the optimization process was concluded (Fang et al., 2013).

### 2.5 Modeling and evaluation

The SVR model (Tan and Zhang, 2016) was constructed utilizing functions from the Python *sklearn* library. The optimization of hyperparameters was executed through 5-fold cross-validation (5-CV), including kernel functions, *C*, and *γ*. The predictive performance of the model was evaluated using the mean square error (*MSE*) (Yuan et al., 2007), calculated by the formula below (Equation 1):


(1) $$\mathrm{MSE}=\frac{{\left(y_{i}-{\hat{y}}_{i}\right)}^{2}}{n}$$

Here, $y_{i}$ , ${\hat{y}}_{i}$ and *n* are the observed value, predicted value and sample size, respectively. Based on the 5-CV strategy, model optimization was performed over different kernel functions and the hyperparameters *C* and γ. The candidate kernel types included *linear*, *polynomial*, *radial basis function* (*RBF*), and *sigmoid*. The penalty parameter *C* was searched within the range [2^−5^, 2^15^], and the kernel coefficient γ was varied from 2^−15^ to 2^3^. The hyperparameter tuning was conducted using the *GridSearchCV* function from the Python package *scikit-learn*. Lower *MSE* scores across the parameter grid indicate better predictive performance of the corresponding hyperparameter combination.

### 2.6 Nonlinear feature screening

Given a dataset D = {*y_i_*, *x_ij_*} (*i* = 1, 2, …, *n*; *j* = 1, 2, …, *m*), containing *n* samples (treatments) and *m* features (factors), we first performed 5-CV with SVR using all *m* features to compute the initial cross-validation *MSE*, denoted as *MSE*_0_. Subsequently, for each feature *j*, we removed that feature and performed the 5-CV to obtain *MSE_j_* (*j* = 1, 2, …, *m*). If the minimum value in the set of *MSE_j_* is smaller than the initial *MSE* (*MSE*_0_), the feature corresponding to the minimum *MSE_j_*, is removed, and the next round of screening proceeds. Otherwise, the feature selection process terminates.

### 2.7 Full combination prediction and frequency statistical optimization

Through a nonlinear feature screening process, features with minimal influence on PQQ yield were eliminated to reduce the impact of irrelevant factors on model performance. Subsequently, an SVR prediction model was constructed using only the retained factors, with the optimal kernel function and hyperparameters determined via 5-CV. All possible treatment combinations based on the levels of the retained factors were then generated and used as inputs to the trained model, yielding predicted PQQ production values. Based on these predictions, treatments with high expected yields (using a threshold of 50 mg/L in this study) were selected. The frequency of occurrence of each factor level among these high-yield treatments was then analyzed; a higher frequency suggests a greater likelihood that the corresponding level contributes to enhanced PQQ production. If the most frequent level of a given factor coincides with the upper or lower bound of its tested range, this implies that the optimal level may lie beyond the current range, warranting a subsequent round of uniform design experiments with an expanded level range in the indicated direction.

## 3 Results

### 3.1 Initial fermentation medium and the upper and lower limits of each factor

Utilizing the fermentation parameters delineated in the methodology sections (2.1) as the foundational framework, the PQQ production for the *A. calcoaceticus* stood at 43.65 mg/L. Initial experimental findings underscored the substantial impact of eight factors—namely, yeast powder, anhydrous ammonium sulfate, L-glutamic acid, L-tyrosine, calcium chloride quantities within the fermentation composition, along with the inoculation volume, fermentation temperature, and pH levels—on the PQQ production of the *A. calcoaceticus*. Consequently, this investigation proceeded to refine these eight key factors through the utilization of UD-SVR. The stipulated ranges for each factor were delineated in Table 1, while the outcomes of the initial 40 systematically designed treatments (N1 ~ N40) by UD and their corresponding PQQ production were detailed in Supplementary Table S1. Within this cohort of 40 treatments, the pinnacle PQQ production reached 65.56 mg/L, marking a notable 50% escalation compared to the foundational formula. Notably, five treatments exhibited production surpassing 50 mg/L, representing 12.5% of all treatments, while 13 treatments demonstrated production exceeding 43 mg/L, representing 32.5%. The lowest PQQ production recorded was 10.12 mg/L.

**Table 1:** The predetermined ranges for each factor in the initial optimization phase.

Factor levels	Yeast powder *x*_1_ (g/L)	Anhydrous ammonium sulfate *x*_2_ (g/L)	L-glutamic acid *x*_3_ (g/L)	L-tyrosine *x*_4_ (g/L)	Calcium chloride *x*_5_ (g/L)	Temperature *x*_6_ (°C)	Inoculum volume *x*_7_ (%)	pH *x*_8_
L1	7	1.0	0.5	0.5	0.2	26	0.1	6.1
L2	10	1.5	1.0	1.0	0.3	27	0.3	6.3
L3	13	2.0	1.5	1.5	0.4	28	0.5	6.5
L4	16	2.5	2.0	2.0	0.5	29	0.7	6.7
L5	19	3.0	2.5	2.5	0.6	-	0.9	6.9

### 3.2 Optimal kernel function selection and nonlinear factor screening results

Using PQQ production data from the initial set of 40 treatments as the dependent variable (*y*), and the eight factors slated for optimization as independent variables (*x*_1_-*x*_8_), the RBF emerged as the optimal choice following SVR and the criterion of minimizing *MSE* during a 5-CV. Additionally, after a process of nonlinear factor screening, six key factors were singled out: the levels of yeast powder (*x*_1_), anhydrous ammonium sulfate (*x*_2_), L-glutamic acid (*x*_3_), L-tyrosine (*x*_4_), as well as the fermentation temperature (*x*_6_) and pH level (*x*_8_). Within the prescribed ranges for each factor as outlined in Table 1, the impact of calcium chloride (*x*_5_) content and inoculation volume (*x*_7_) on PQQ production was deemed negligible. Details of the factor screening procedure were elaborated in Table 2.

**Table 2:** Nonlinear factor screening process and *MSE* values.

Screening rounds	Baseline *MSE*	*x*_1_	*x*_2_	*x*_3_	*x*_4_	*x*_5_	*x*_6_	*x*_7_	*x*_8_	Excluded factors
1	24.42	23.20	22.29	22.47	22.43	21.51	22.10	21.47	22.14	*x*_7_
2	21.47	22.20	21.72	22.60	22.84	19.97	21.36	-	21.31	*x*_5_
3	19.97	20.81	20.02	21.15	22.41	-	21.16	-	20.80	-

### 3.3 Frequency statistical optimization through full combination prediction

Through meticulous feature selection, a new dataset was curated, featuring 6 input characteristics and 40 samples. The RBF was chosen as the optimal function, with hyperparameters *C* and *γ* fine-tuned through grid search to construct an SVR training model. The resulting model achieved a 5-CV MSE of 19.97 and a coefficient of determination (*R*^2^) of 0.88 (Supplementary Figure S2), indicating strong predictive performance. Subsequently, predictions were generated for the PQQ production across all 125,000 comprehensive combinations of *x*_1_ (5 levels), *x*_2_ (5 levels), *x*_3_ (5 levels), *x*_4_ (5 levels), *x*_6_ (4 levels), and *x*_8_ (5 levels).

**Figure 1:**
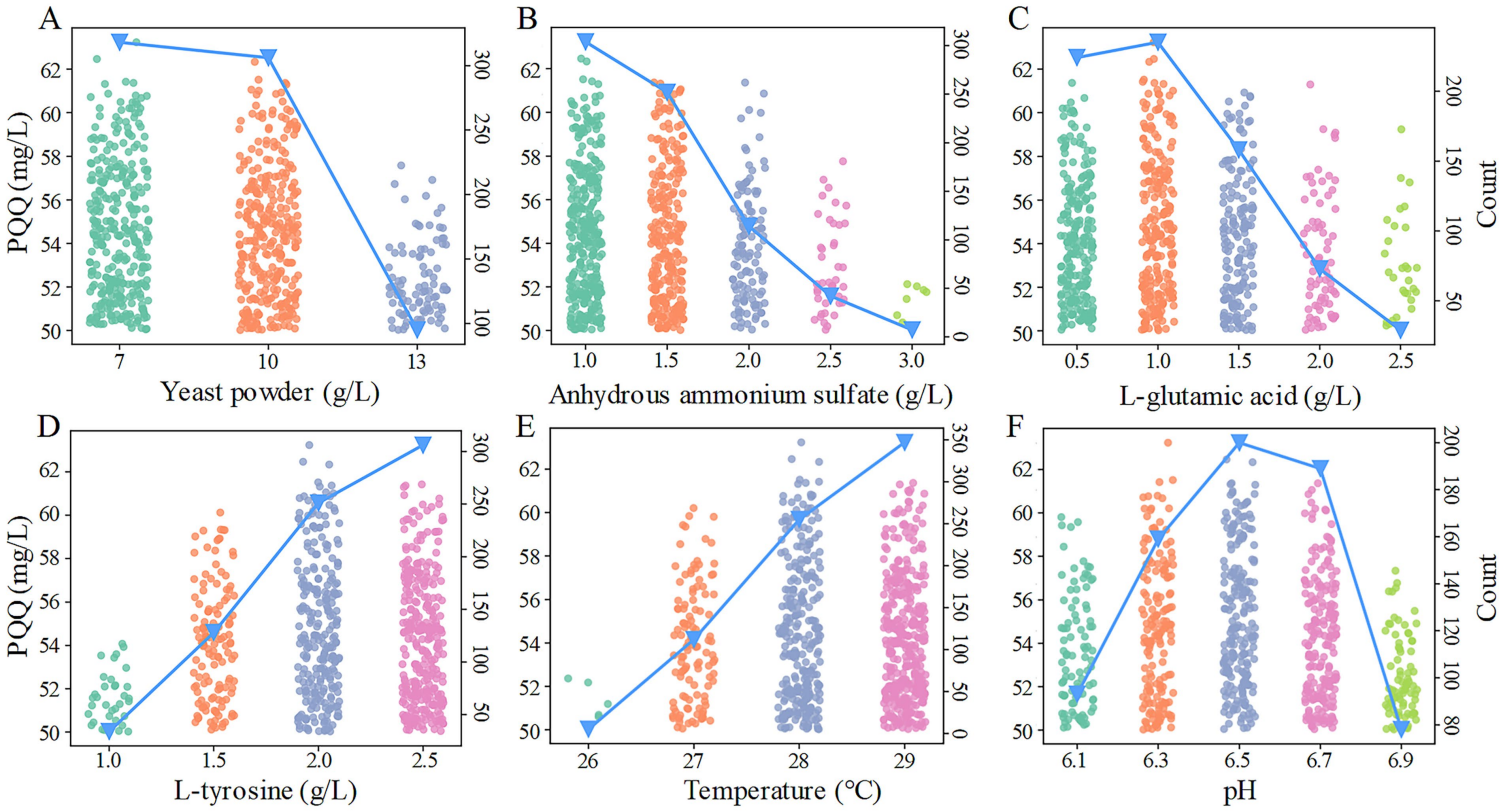
Frequency distribution of the six retained factors. **(A–F)** represent the frequency distributions of yeast powder, anhydrous ammonium sulfate, L-glutamic acid, L-tyrosine, temperature, and pH, respectively. The left *y*-axis of each subgraph corresponds to the predicted PQQ production values (scatterplot), while the right *y*-axis represents the frequency of occurrence for each level (blue line plot).

The average prediction among the 125,000 values stands at 33.92 mg/L, with the highest reaching 63.24 mg/L and the lowest at 10.86 mg/L. Notably, 719 instances surpass the 50 mg/L in these forecasts. Subsequently, a thorough examination of the frequency distribution of each retained factor’s levels was conducted based on these instances (Figure 1). In scenarios where the prediction exceeds 50 mg/L, a predominant pattern emerges concerning yeast powder usage: instances featuring 7 mg/L and 10 mg/L are prevalent, with 318 and 306 occurrences respectively, while instances with 13 mg/L of yeast powder are notably fewer at 95. This observation underscores that an increase in yeast powder quantity does not necessarily translate to improved outcomes. Consequently, for the forthcoming optimization phase, a reduction in yeast powder quantity warrants exploration. The trajectory of anhydrous ammonium sulfate usage mirrors that of yeast powder, signaling a necessity for further reduction in its utilization during the subsequent optimization iteration. Regarding L-glutamic acid, there is a trend towards decreased usage, peaking at 1.0 g/L (235 occurrences), with a notable presence of 224 occurrences at 0.5 g/L as well. While no extrapolation is imperative in the next optimization cycle, it is crucial to consider both the 0.5 g/L and 1.0 g/L levels. Contrary to the trends observed in anhydrous ammonium sulfate and L-glutamic acid, the trend in L-tyrosine usage necessitates extrapolation towards increased quantities. Despite the instances with a L-tyrosine usage of 2.0 g/L (251 occurrences) being fewer than those at 2.5 mg/L (306 occurrences), the highest predicted value corresponds to a L-tyrosine usage of 2.0 g/L. Hence, for the subsequent optimization phase, extrapolation based on the 2.0 g/L and 2.5 g/L levels is advised. The temperature trend aligns with the pattern observed in L-tyrosine usage, warranting extrapolation based on 28°C and 29°C in the forthcoming optimization cycle. Notably, a conspicuous peak is evident at a pH value of 6.5, suggesting that it can be fixed at 6.5 without necessitating further extrapolation.

### 3.4 Second-round uniform design

Informed by the initial round of factor screening and statistical frequency optimization, three factors—calcium chloride, inoculum volume, and pH—have been earmarked for stabilization. Notably, calcium chloride and inoculum volume, lacking in retained significance, are consequently anchored at their median values of 0.4 g/L and 0.4%, respectively. The pH level, as per the statistical frequency optimization outcomes, is set at a fixed value of 6.5. Subsequent to the statistical frequency optimization findings, yeast powder, anhydrous ammonium sulfate, L-glutamic acid, L-tyrosine, and fermentation temperature—comprising a total of five factors—have been utilized to craft a uniform design of 26 treatments across 2–3 levels. The specific ranges for each factor are meticulously outlined in Table 3, while the intricate particulars of every treatment and the corresponding quantified PQQ production are catalogued in Supplementary Table S2.

**Table 3:** Predefined ranges for each factor in the second round of optimization.

Factor levels	Yeast powder *x*_1_ (g/L)	Anhydrous ammonium sulfate *x*_2_ (g/L)	L-glutamic acid *x*_3_ (g/L)	L-tyrosine *x*_4_ (g/L)	Temperature *x*_6_ (°C)
L1	5	0.5	0.5	2.0	28
L2	7	1.0	1.0	2.5	29
L3	9	-	-	3.0	30

Within the cohort of 26 treatments during the subsequent phase, the pinnacle production peaks at 71.42 mg/L, representing a notable 63.62% surge in contrast to the foundational formulation. This pinnacle attainment aligns with yeast powder at 5.0 g/L, anhydrous ammonium sulfate at 1 g/L, L-glutamic acid at 0.5 g/L, L-tyrosine at 2.0 g/L, and a constant temperature of 30°C. Noteworthy is the presence of 15 groups surpassing the 50.0 mg/L production, constituting 57.69% of the aggregate, alongside 22 groups surpassing 43.0 mg/L, accounting for 84.62%. The treatment production with the lowest PQQ production registers at 30.81 mg/L. Evidently, subsequent to the initial optimization phase, a substantial upsurge in PQQ production is discernible across each treatment in the ensuing round (*p* value of *t*-test is 2.3043e-05).

### 3.5 Full combination prediction and optimal combination verification

Utilizing the 26 treatments as the training set from the second round experiment, the radial basis kernel emerged as the optimal choice for the kernel function. Following meticulous grid optimization, the pivotal hyperparameters *C* and *γ* were pinpointed, culminating in the construction of a robust SVR training model. Forecasts were made for PQQ production across all 108 comprehensive treatment combinations, encompassing yeast powder (3 levels), anhydrous ammonium sulfate (2 levels), L-glutamic acid (2 levels), L-tyrosine (3 levels), and fermentation temperature (3 levels). The zenith of these predictions peaked at 70.25 mg/L, linked to yeast powder at 5 g/L, anhydrous ammonium sulfate at 0.5 g/L, L-glutamic acid at 0.5 g/L, L-tyrosine at 2 g/L, and a temperature set at 30°C. This formula harmonized closely with the optimal blend derived from the second-round uniform design, with the sole alteration being the decrease in anhydrous ammonium sulfate from 1.0 g/L to 0.5 g/L, underscoring the model’s robust predictive capacity. Subsequent experimental validation of this formula production a notable PQQ production of 73.40 mg/L, showcasing a remarkable 68.15% enhancement compared to the standard formulation.

## 4 Conclusion

Optimization of a complex 8-factor formulation was meticulously undertaken using the UD-SVR method across two rounds of uniform design, encompassing a total of 66 treatments. The measured PQQ content surged impressively from the initial scheme’s 43.65 mg/L to a notable 73.40 mg/L. The refined formulation tailored for PQQ production by *A. calcoaceticus* featured precise fermentation conditions: yeast powder at 5 g/L, anhydrous ammonium sulfate at 0.5 g/L, L-glutamic acid at 0.5 g/L, L-tyrosine at 2.0 g/L, Na_2_HPO_4_ at 2.0 g/L, KH_2_PO_4_ at 1.4 g/L, MgSO_4_·7H_2_O at 1.0 g/L, and calcium chloride at 0.4 g/L. Essential trace elements were meticulously included: FeSO4·7H2O at 80.0 mg/L, ZnSO_4_·7H_2_O at 22.5 mg/L, KI at 0.3 mg/L, H_3_BO_3_ at 3.0 mg/L, CuSO_4_ at 5.0 mg/L, NaCl at 15.0 mg/L, maintaining a pH of 6.5. The inoculation rate stood at 0.5%, with a fermentation temperature set at 30°C and a duration of 7 days.

In comparison to the initial formulation, the optimized scheme not only substantially amplified PQQ production from *A. calcoaceticus* but also effectively curtailed fermentation costs. Noteworthy adjustments included halving the yeast powder requirement from 10.0 g/L to 5.0 g/L, marking a significant 50% reduction. Furthermore, the initial formulation, demanding a total nitrogen source of 4.0 g/L (anhydrous ammonium sulfate: L-glutamic acid: L-tyrosine = 2:1:1), was refined in the optimized scheme to a more efficient 3.0 g/L ratio (0.5:0.5:2). The optimized strategy also challenged the nitrogen source ratio derived from single-factor experiments by elevating the proportion of L-tyrosine.

This study primarily honed the PQQ production capacity of *A. calcoaceticus* at the laboratory flask level. Future pursuits should encompass the integration of mutagenesis techniques to cultivate high-production strains and further refine fermentation conditions at the bioreactor and industrial scales to augment PQQ production capacity.

## Data Availability

The original contributions presented in the study are included in the article/Supplementary material, further inquiries can be directed to the corresponding authors.
