# Supplementary material for: Enhancing PQQ production in Acinetobacter calcoaceticus through uniform design and support vector regression
Source: Front Microbiol. 2025 Aug 7;16:1556322. doi: 10.3389/fmicb.2025.1556322 (PMC12367485; doi:10.3389/fmicb.2025.1556322)
Supplement: Supplementary file 1 [file Table_1.docx]

Table S1 The uniform design treatments (N1~N40) and the measured PQQ production

| Treatments | *x*_1_(g/L) | *x*_2_(g/L) | *x*_3_(g/L) | *x*_4_(g/L) | *x*_5_(g/L) | *x*_6_(℃) | *x*_7_(%) | *x*_8_ | PQQ production (mg/L) |
| --- | --- | --- | --- | --- | --- | --- | --- | --- | --- |
| N1 | 16 | 1.0 | 2.0 | 0.5 | 0.3 | 26 | 0.3 | 6.7 | 29.688±  1.653 |
| N2 | 7 | 3.0 | 2.5 | 0.5 | 0.5 | 28 | 0.7 | 6.7 | 47.712 ±4.079 |
| N3 | 7 | 2.0 | 1.0 | 1.0 | 0.2 | 26 | 0.1 | 6.5 | 11.204±  1.837 |
| N4 | 16 | 3.0 | 1.5 | 1.0 | 0.4 | 27 | 0.1 | 6.9 | 32.675 ±  4.194 |
| N5 | 13 | 1.5 | 2.5 | 1.0 | 0.2 | 28 | 0.5 | 6.1 | 35.605 ±  5.108 |
| N6 | 7 | 1.5 | 1.0 | 2.5 | 0.3 | 27 | 0.5 | 6.1 | 64.724±  3.575 |
| N7 | 16 | 2.0 | 2.5 | 2.0 | 0.2 | 26 | 0.9 | 6.9 | 40.482±  6.819 |
| N8 | 13 | 1.0 | 1.0 | 1.5 | 0.6 | 27 | 0.1 | 6.7 | 51.189 ±  0.524 |
| N9 | 10 | 1.5 | 0.5 | 1.0 | 0.6 | 27 | 0.7 | 6.3 | 50.106 ±  1.571 |
| N10 | 16 | 1.5 | 0.5 | 2.0 | 0.2 | 29 | 0.1 | 6.3 | 38.029 ±  3.077 |
| N11 | 16 | 2.0 | 0.5 | 2.0 | 0.5 | 28 | 0.9 | 6.1 | 22.863±  2.188 |
| N12 | 19 | 2.0 | 0.5 | 0.5 | 0.6 | 26 | 0.5 | 6.5 | 10.121 ±  1.726 |
| N13 | 13 | 1.0 | 0.5 | 0.5 | 0.4 | 28 | 0.5 | 6.9 | 32.041 ±  3.833 |
| N14 | 16 | 3.0 | 1.0 | 2.5 | 0.5 | 28 | 0.3 | 6.5 | 38.996±  3.375 |
| N15 | 7 | 1.0 | 1.5 | 1.5 | 0.2 | 28 | 0.9 | 6.3 | 62.040 ±  3.368 |
| N16 | 10 | 2.5 | 2.0 | 0.5 | 0.2 | 27 | 0.3 | 6.3 | 44.551 ±  3.858 |
| N17 | 16 | 2.5 | 2.5 | 2.0 | 0.6 | 27 | 0.5 | 6.7 | 43.282±  1.286 |
| N18 | 19 | 1.0 | 1.0 | 2.5 | 0.3 | 27 | 0.7 | 6.5 | 31.853±  7.922 |
| N19 | 19 | 2.5 | 1.0 | 1.0 | 0.4 | 28 | 0.3 | 6.1 | 12.560 ±  2.168 |
| N20 | 19 | 3.0 | 2.0 | 1.5 | 0.3 | 29 | 0.7 | 6.1 | 30.901 ±  5.350 |
| N21 | 19 | 1.5 | 2.5 | 1.5 | 0.5 | 26 | 0.3 | 6.3 | 13.325 ±  5.170 |
| N22 | 10 | 2.5 | 1.0 | 1.0 | 0.6 | 29 | 0.9 | 6.9 | 46.773 ±  2.692 |
| N23 | 13 | 3.0 | 1.0 | 0.5 | 0.3 | 26 | 0.9 | 6.3 | 29.125 ±  7.005 |
| N24 | 13 | 2.5 | 1.5 | 2.5 | 0.2 | 29 | 0.5 | 6.7 | 62.011 ±  2.013 |
| N25 | 19 | 1.5 | 1.5 | 0.5 | 0.4 | 29 | 0.9 | 6.7 | 21.333±  5.106 |
| N26 | 7 | 3.0 | 1.5 | 2.0 | 0.6 | 29 | 0.5 | 6.3 | 41.967 ±  1.439 |
| N27 | 10 | 1.0 | 2.5 | 2.5 | 0.4 | 29 | 0.3 | 6.5 | 65.562 ±  1.938 |
| N28 | 13 | 2.5 | 2.0 | 2.5 | 0.5 | 27 | 0.9 | 6.3 | 33.469 ±  2.765 |
| N29 | 13 | 2.5 | 2.5 | 1.5 | 0.3 | 28 | 0.1 | 6.5 | 36.456 ±  8.161 |
| N30 | 10 | 1.5 | 2.0 | 2.0 | 0.3 | 28 | 0.7 | 6.9 | 45.893 ±  1.481 |
| N31 | 16 | 1.0 | 2.0 | 1.0 | 0.5 | 29 | 0.7 | 6.5 | 39.963 ±  2.562 |
| N32 | 10 | 3.0 | 2.0 | 2.0 | 0.4 | 26 | 0.1 | 6.1 | 51.579 ±  6.993 |
| N33 | 10 | 2.5 | 0.5 | 2.5 | 0.4 | 26 | 0.7 | 6.7 | 38.606 ±  4.451 |
| N34 | 7 | 1.5 | 1.5 | 2.0 | 0.5 | 26 | 0.3 | 6.9 | 44.292 ±  1.909 |
| N35 | 13 | 1.0 | 1.5 | 1.5 | 0.6 | 26 | 0.7 | 6.1 | 38.274±  3.010 |
| N36 | 10 | 2.0 | 1.5 | 0.5 | 0.5 | 29 | 0.1 | 6.1 | 39.631 ±  4.756 |
| N37 | 7 | 2.0 | 2.5 | 1.0 | 0.4 | 27 | 0.9 | 6.5 | 25.792±  4.869 |
| N38 | 19 | 2.0 | 2.0 | 2.5 | 0.6 | 28 | 0.1 | 6.9 | 22.575±  1.999 |
| N39 | 7 | 2.0 | 0.5 | 1.5 | 0.3 | 29 | 0.3 | 6.7 | 65.244 ±  1.048 |
| N40 | 19 | 3.0 | 0.5 | 1.5 | 0.2 | 27 | 0.5 | 6.9 | 26.903 ±  2.242 |

*x*_1_: Yeast powder; *x*_2_: Anhydrous ammonium sulfate; *x*_3_: L-glutamic acid; *x*_4:_ L-tyrosine; *x*_5_: Calcium chloride; *x*_6_: Temperature; *x*_7_: Inoculum volume; *x*_8_: pH

Table S2 Second-round uniform design and the measured PQQ production

| Treatments | *x*_1_(g/L) | *x*_2_(g/L) | *x*_3_(g/L) | *x*_4_(g/L) | *x*_5_(g/L) | *x*_6_(℃) | *x*_7_(%) | *x*_8_ | PQQ production (mg/L) |
| --- | --- | --- | --- | --- | --- | --- | --- | --- | --- |
| N1 | 9 | 0.5 | 1.0 | 3.0 | 0.4 | 29 | 0.5 | 6.5 | 66.629±  2.851 |
| N2 | 9 | 0.5 | 0.5 | 2.0 | 0.4 | 28 | 0.5 | 6.5 | 55.576±  6.883 |
| N3 | 9 | 1.0 | 0.5 | 3.0 | 0.4 | 28 | 0.5 | 6.5 | 44.089±  4.227 |
| N4 | 9 | 1.0 | 1.0 | 2.5 | 0.4 | 30 | 0.5 | 6.5 | 56.167±  6.184 |
| N5 | 7 | 0.5 | 1.0 | 2.5 | 0.4 | 28 | 0.5 | 6.5 | 44.364±  6.917 |
| N6 | 9 | 1.0 | 1.0 | 2.5 | 0.4 | 28 | 0.5 | 6.5 | 55.807±  6.218 |
| N7 | 5 | 1.0 | 0.5 | 2 | 0.4 | 30 | 0.5 | 6.5 | 71.420±  11.236 |
| N8 | 5 | 0.5 | 1.0 | 2.5 | 0.4 | 30 | 0.5 | 6.5 | 51.506±  3.488 |
| N9 | 7 | 1.0 | 1.0 | 3.0 | 0.4 | 29 | 0.5 | 6.5 | 58.115±  0.900 |
| N10 | 7 | 1.0 | 0.5 | 3.0 | 0.4 | 29 | 0.5 | 6.5 | 56.211±  5.319 |
| N11 | 7 | 1.0 | 1.0 | 2.0 | 0.4 | 29 | 0.5 | 6.5 | 64.912±  5.249 |
| N12 | 5 | 0.5 | 0.5 | 2.0 | 0.4 | 29 | 0.5 | 6.5 | 57.452±  6.461 |
| N13 | 7 | 1.0 | 0.5 | 2.5 | 0.4 | 30 | 0.5 | 6.5 | 49.169±  7.686 |
| N14 | 5 | 1.0 | 1.0 | 3.0 | 0.4 | 30 | 0.5 | 6.5 | 43.267±  4.576 |
| N15 | 5 | 0.5 | 0.5 | 3.0 | 0.4 | 29 | 0.5 | 6.5 | 41.665±  6.430 |
| N16 | 9 | 0.5 | 1.0 | 2.0 | 0.4 | 30 | 0.5 | 6.5 | 48.851±  8.545 |
| N17 | 7 | 0.5 | 0.5 | 2.5 | 0.4 | 28 | 0.5 | 6.5 | 58.505±  11.724 |
| N18 | 7 | 0.5 | 1.0 | 2.0 | 0.4 | 29 | 0.5 | 6.5 | 68.447±  6.570 |
| N19 | 9 | 1.0 | 0.5 | 2.0 | 0.4 | 29 | 0.5 | 6.5 | 63.094±  1.669 |
| N20 | 5 | 1.0 | 0.5 | 2.5 | 0.4 | 28 | 0.5 | 6.5 | 45.908±  10.054 |
| N21 | 5 | 0.5 | 1.0 | 3.0 | 0.4 | 28 | 0.5 | 6.5 | 39.703±  4.784 |
| N22 | 7 | 0.5 | 0.5 | 2.5 | 0.4 | 30 | 0.5 | 6.5 | 47.120±  7.027 |
| N23 | 9 | 0.5 | 0.5 | 3.0 | 0.4 | 30 | 0.5 | 6.5 | 56.874±  9.797 |
| N24 | 5 | 1.0 | 1.0 | 2.0 | 0.4 | 28 | 0.5 | 6.5 | 30.814±  6.873 |
| N25 | 10 | 1.0 | 0.5 | 2.5 | 0.4 | 29 | 0.5 | 6.7 | 64.782±  1.668 |
| N26 | 7 | 1.0 | 1.0 | 2.0 | 0.4 | 28 | 0.5 | 6.3 | 33.339±  2.519 |
