# Supplementary figures and images for: Enhancing PQQ production in Acinetobacter calcoaceticus through uniform design and support vector regression
Source: Front Microbiol. 2025 Aug 7;16:1556322. doi: 10.3389/fmicb.2025.1556322 (PMC12367485; doi:10.3389/fmicb.2025.1556322)

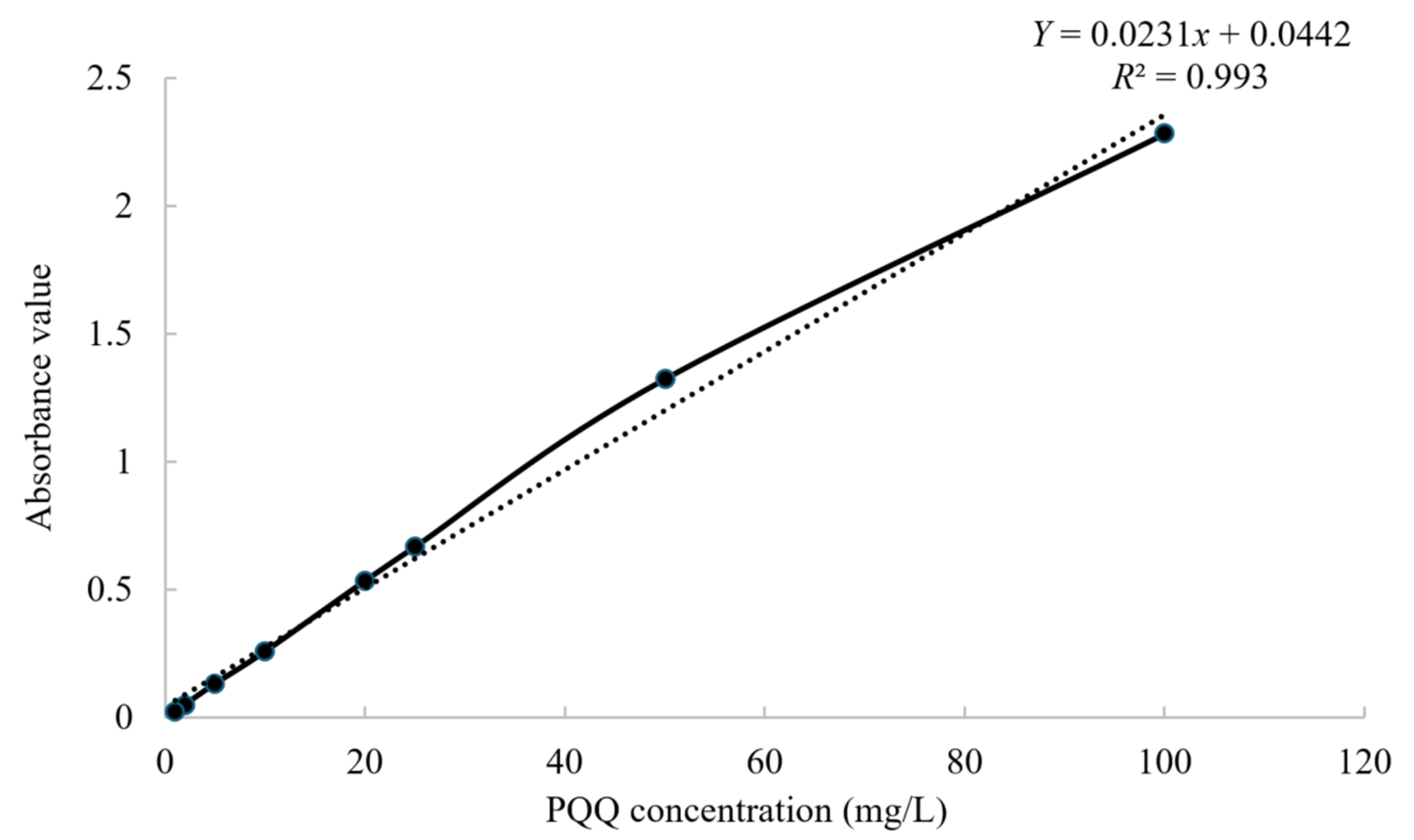

Supplement: Supplementary file 2 [file Image_1.tif]

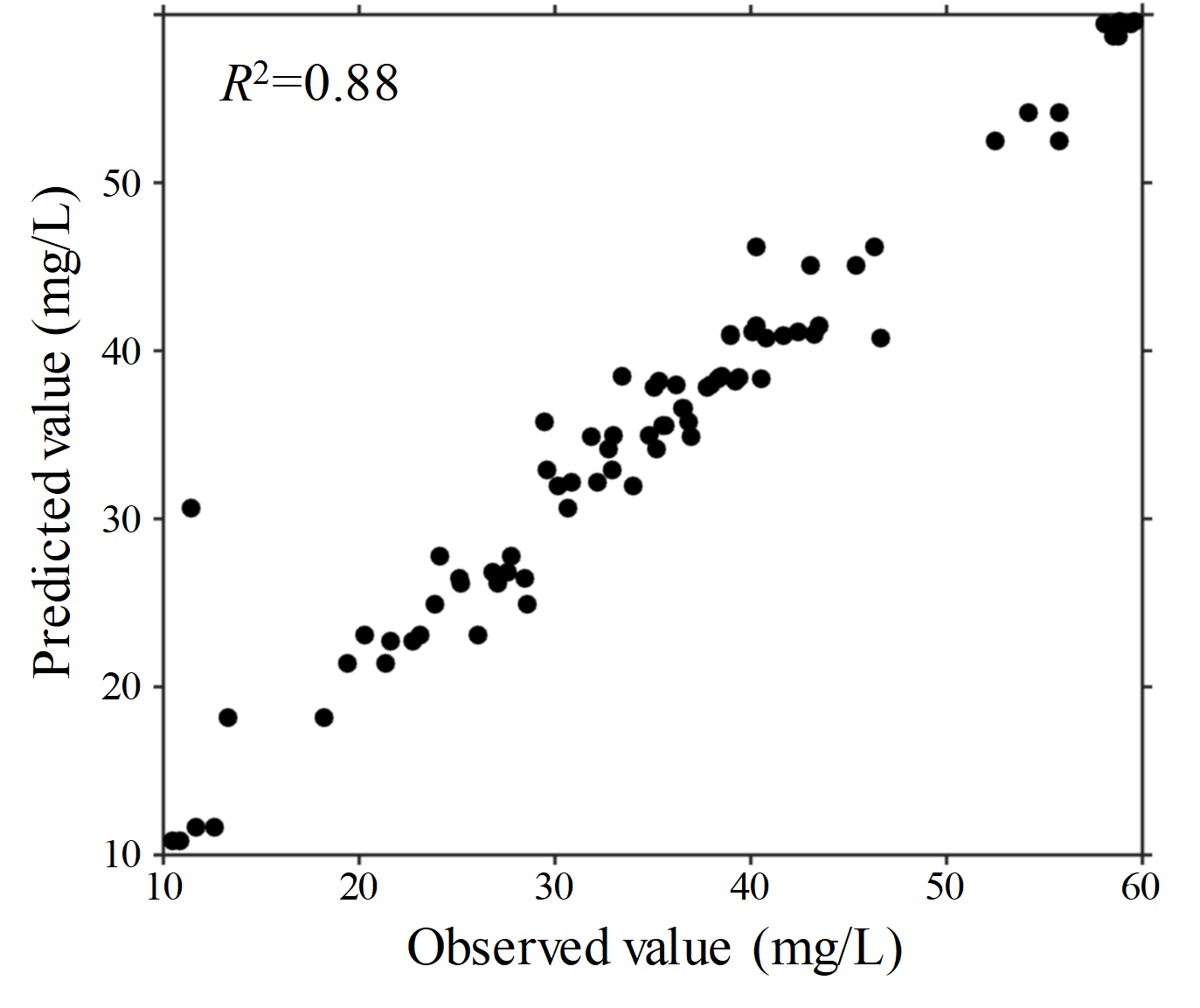

Supplement: Supplementary file 3 [file Image_2.tif]
